# Cerebroplacental Ratio in Monochorionic Diamniotic Twin Pregnancies with and Without Gestational Diabetes: A Longitudinal Cohort Study

**DOI:** 10.3390/jcm15103864

**Published:** 2026-05-17

**Authors:** Gülen Yerlikaya-Schatten, Marija Adamovic, Anja Catic, Kitana Hendling, Vivien Sauer, Stephanie Springer, Florian Heinzl, Theresa Reischer

**Affiliations:** Department of Obstetrics and Gynecology, Division of Obstetrics and Feto-Maternal Medicine, Medical University of Vienna, 1090 Vienna, Austria; guelen.yerlikaya-schatten@meduniwien.ac.at (G.Y.-S.);

**Keywords:** twin pregnancy, gestational diabetes, cerebroplacental ratio, Doppler assessment

## Abstract

**Objective:** To investigate whether gestational diabetes mellitus (GDM), including insulin-treated GDM, affects cerebroplacental ratio (CPR) values in monochorionic diamniotic (MCDA) twin pregnancies. **Methods:** This retrospective single-center cohort study included a total of 262 MCDA twin pregnancies managed at a tertiary referral center, comprising pregnancies without GDM (n = 120), with diet-controlled GDM (n = 80), and with insulin-treated GDM (n = 62). Doppler ultrasound examinations were performed at three gestational time points between 24 and 36 weeks of gestation. CPR, umbilical artery pulsatility index (UA-PI), and middle cerebral artery pulsatility index (MCA-PI) were compared longitudinally between groups. Doppler indices were compared between groups without adjustment for baseline differences such as BMI and parity **Results:** Maternal body mass index was significantly higher in pregnancies complicated by GDM, particularly in those requiring insulin therapy (*p* < 0.001). Estimated fetal weight was higher in the insulin-treated GDM group at mid-gestation (28–32 weeks; *p* = 0.01). However, CPR values remained within normal ranges throughout all screening points across all three groups. No relevant differences in UA-PI, MCA-PI, gestational age at delivery, Apgar scores, or umbilical cord pH were observed between groups. **Conclusions:** In MCDA twin pregnancies, gestational diabetes—regardless of insulin treatment—does not appear to significantly influence cerebroplacental ratio values throughout gestation. No statistically significant differences in CPR values were observed between groups. No statistically significant differences in CPR values were detected between groups. However, given the exploratory design and lack of adjustment for confounders, subtle effects cannot be excluded. The clinical utility of CPR in this population requires further investigation.

## 1. Introduction

The cerebro-placental ratio (CPR) reflects the degree of increased cerebral perfusion that occurs in response to fetal hypoxia and is linked to various adverse perinatal outcomes and is mostly used to assess fetal well-being, especially in cases of fetal growth restriction (FGR) and can help determine the optimal time for delivery to minimize risks. Therefore, CPR has become a valuable clinical tool, offering better predictive value for adverse outcomes than Doppler measurements of the middle cerebral or umbilical arteries alone, as it reflects both suboptimal placental function and fetal cardiovascular compensation [1,2]. In twin pregnancies, interpretation is more complex due to differences in placental sharing and inter-twin variability. General twin pregnancies, especially monochorionic twins, are at higher risk of adverse outcomes compared to dichorionic twin and singleton pregnancies [3]. In MCDA twin pregnancies, placental vascular anastomoses may buffer inter-fetal hemodynamic differences and could potentially stabilize Doppler indices such as CPR despite maternal metabolic disturbances. Gestational diabetes mellitus (GDM) is associated with metabolic disturbances that can affect placental function and fetal growth [4]. From a biological perspective, GDM—particularly when requiring insulin therapy as a proxy of increased metabolic burden—may influence fetoplacental circulation, potentially resulting in altered MCA-PI, UA-PI, and consequently CPR values or trajectories over gestation. Although research on the performance of CPR in diabetic pregnancies is limited, emerging evidence indicates that a low CPR (below the 10th percentile), regardless of GDM treatment, is associated with poorer perinatal outcomes, including increased rates of low birth weight and preterm birth [5]. On the contrary, a recent work by Cardinali et al. observed that CPR can indeed be associated with adverse perinatal outcomes, but it fails as a general screening tool for adverse outcomes in singleton pregnancies complicated by GDM [6]. However, this may be different in twin pregnancies with GDM. The presence of GDM may increase the risks already inherent in twin pregnancies, especially in monochorionic twins, leading to a higher likelihood of complications and adverse perinatal outcomes, even in the absence of FGR. Although various cut-offs have been proposed for defining an abnormal CPR value, there is currently insufficient evidence to support the use of one specific cut-off over another in both singleton and twin pregnancies [7].

Therefore, this study aimed to investigate whether GDM status (including insulin-treated GDM as a marker of disease severity) is associated with altered CPR levels or longitudinal trajectories in MCDA twin pregnancies.

## 2. Materials and Methods

This was a retrospective cohort study in a tertiary referral center. The study period spanned from 2003 to 2022. A total of 262 monochorionic-diamniotic (MCDA) twin pregnancies with (n = 142) and without (n = 120) GDM were included. Monoamniotic monochorionic twins were excluded, as were MCDA with twin-to-twin transfusion syndrome (TTTS). Overall, 62 women needed insulin treatment and 80 were managed by diet alone. The diagnosis of GDM was determined using a standardized 75 g oGTT at 24–28 weeks of gestation [8]. In cases with known risk factors (especially those with a previous history of GDM), testing for diabetes was performed earlier. The diagnostic thresholds for GDM were defined according to the IADPSG criteria after 2010 as follows: fasting blood glucose level ≥ 92 mg/dL, 1 h plasma glucose level ≥ 180 mg/dL, and 2 h plasma glucose level ≥ 153 mg/dL [8]. Before 2010, our cut-off values for the diagnosis of GDM were fasting blood glucose level ≥ 95 mg/dL, 1 h plasma glucose level ≥ 180 mg/dL, and 2 h plasma glucose level ≥ 155 mg/dL [8]. Additionally, 18 of 262 patients were evaluated before the new criteria were applied. The majority of patients therefore were diagnosed using the criteria of the International Association of Diabetes and Pregnancy Study Groups (IADPSG) for diagnosing gestational diabetes mellitus (GDM) using a one-step 75 g oral glucose tolerance test (OGTT). Before Austria adopted the IADPSG criteria, screening and diagnosis of GDM were less standardized. Typically, a two-step approach was used, starting with a screening test (like a glucose challenge test), followed by an oral glucose tolerance test (OGTT) if abnormal. However, all patients were instructed to undergo autonomous capillary blood glucoses monitoring and educated about glycemic treatment targets. Follow-up visits were scheduled biweekly, during which blood glucose charts were reviewed alongside biometric ultrasound and Doppler measurements to assess fetal growth percentiles and amniotic fluid levels. Lifestyle changes were the initial approach, including personalized plans for nutrition, exercise, and self-monitoring. Pharmacological treatment was started if target glucose levels were not met (fasting: 94 mg/dL and/or >140 mg/dL after meals). In cases with pharmacological treatment of GDM, only those with Insulin therapy were included. Women managed with metformin or a combination of metformin and Insulin have been excluded due to a lack of guidelines in regard to metformin prescription. All cases included in this study met the diagnostic criteria of GDM at the time of their diagnosis. CPR measurements and estimated fetal weight (EFW) were recorded at three time points during pregnancy: 24+0 to 26+6 weeks, 28+0 to 32+6 weeks, and 34+0 to 36+6 weeks of gestation. Doppler studies were performed according to ISUOG Guidelines [9]. The following Doppler parameters were measured: umbilical artery pulsatility index (UA-PI), middle cerebral artery pulsatility index (MCA-PI), and middle cerebral artery peak systolic velocity (MCA-Vmax, m/s). Additionally, the following variables were collected from the medical records: age, gravidity, parity, pre-pregnancy body mass index (BMI), conception, smoking, chorionicity, gestational age at delivery, APGAR at 5 min and pH. Furthermore, OGTT results at fasting (G0), 60 min (G60), and 120 min (G120) were acquired. Patient records were electronically examined with the assistance of the obstetric database PIA Fetal Database software Version 5, (Viewpoint, General Electrics Healthcare, Munich, Germany). Subsequently, the data were retrieved and used for statistical assessment.

### Statistical Analysis

Ordinal data are presented as median and interquartile range (IQR), categorical data as absolute and relative frequencies. Groups have been compared with Kruskal–Wallis (ordinal data), respectively χ^2^-test (categorical data). Correlation between CPR levels of twins (assigned randomly to one of two groups) at all three screenings was assessed using intra-class correlation coefficients and Kendall’s τ. Given the clustering of twins within pregnancies, within-pregnancy correlation was considered in exploratory analyses by first fitting a mixed effects model with pair as random effect and DM status as fixed effects for CPR values. This resulted in a singularity for the variable pair (the estimated effect was 0). Despite this, within-pregnancy clustering cannot be ruled out, which should be considered when interpreting the results. While we chose to drop said variable for subsequent models, in order to still account for possible residual intra-twin effects, Huber–White Sandwich estimators have been utilized for calculating more conservative standard errors and *p*-values. Three models were built. Each modeled CPR at screening 3 as a function of previous measurements. Model 1 used CPR at screening 1, model 2 used CPR at screening 2 and model 3 used both. All three also included the respective gestational age at previous screenings. This was done to maximize power, since not all records were available in all twins. While CPR data was not available in 14.1% (37 cases) at the first screening and 4.2% (11 cases) at the second screening, at the third screening data of all cases were available. Details of missing data are listed in Table S1. CPR measurements and gestational age at screening were represented via restricted cubic splines. Additionally, the interaction between CPR and time of sampling as well as GDM status were used as a predictor. This unadjusted analysis limits the interpretability, including, for example, baseline characteristics, even those for which we observed differences (e.g., BMI, parity, assisted conception) between the three groups (GDM, IGDM, no GDM), as covariates were deemed to not be feasible due to sample size concerns. Given repeated measurements and multiple comparisons across three time points and several Doppler parameters, the results should be interpreted as exploratory; no formal correction for multiple testing was applied. As such, interpretability of the models is limited. A *p*-value below 0.05 is viewed as being statistically significant. The study period was from 2003 until 2022, spanning years before and after 2010; therefore, two diagnostic criteria for GDM were applied. A sensitivity analysis restricted to the post-IADPSG period was not feasible and is acknowledged as a limitation.

The study obtained approval from the local Ethics Committee and was conducted in accordance with the principles outlined in the Declaration of Helsinki.

## 3. Results

A total of 262 MCDA twin pregnancies were included in the analysis, comparing 80 with GDM, 62 women with IGDM, and 120 without GDM.

### 3.1. Maternal Characteristics

There was a statistically significant difference in maternal BMI across the groups (*p* < 0.0001). The highest median BMI was observed in the IGDM group with a BMI of 25.06 (IQR 22.49–28.70), followed by the GDM patients solely managed with diet and lifestyle modification (24.57, IQR 21.99–27.64), with the lowest in the non-GDM group (21.48, IQR 20.20–24.44). Distribution of parity also differed significantly (*p* < 0.0001), with higher parity in the GDM and IGDM groups compared to non-GDM. There were no significant differences in gravidity (*p* = 0.747). Assisted conception was more frequent in the IGDM group compared to non-GDM pregnancies (29% vs. 10.2%; *p* = 0.0065) retrospectively.

### 3.2. Perinatal Outcomes

Gestational age at delivery was comparable across all groups, with deliveries occurring around 36 weeks of gestation (36.4 GDM group, 36.4 IGDM group, 36.5 non-GDM group; *p* = 0.826). No significant differences were observed for 5 min Apgar scores (median Apgar 10 in all groups; *p* = 0.128) or cord blood pH (*p* = 0.139), indicating comparable neonatal outcomes and no substantial variations in acid–base status at birth. Smoking status did not differ significantly (*p* = 0.193), although the highest proportion of smokers was found in the IGDM group with 16.1%, respectively. For more details on characteristics of the study population, see Table 1.

### 3.3. Ultrasound Findings

24+0–26+6 weeks of gestation.

EFW did not differ significantly across groups (*p* = 0.648), nor did UA-PI (*p* = 0.991), MCA-PI; (*p* = 0.688), or CPR (*p* = 0.472).

28+0–32+6 weeks of gestation.

Median EFW was significantly higher in the GDM and IGDM groups compared to the non-GDM group, with values of 1531.5 g, 1613.5 g, and 1481 g, respectively (*p* = 0.01). The distribution of UA-PI, MCA-PI, and CPR values showed no statistically significant differences among the three groups (*p* > 0.19).

34+0–36+6 weeks of gestation.

**Figure 1:**
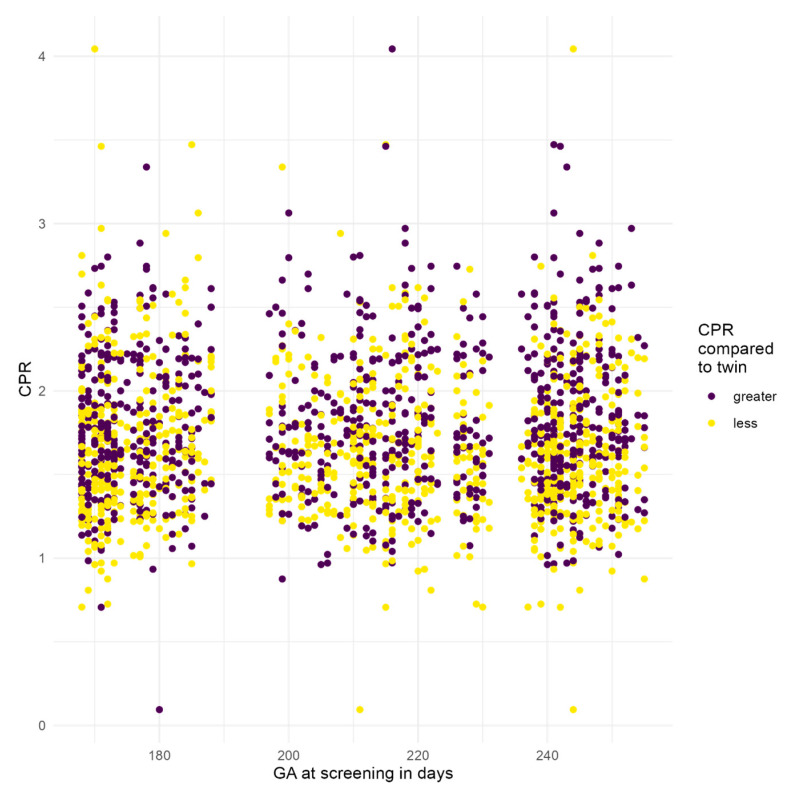
Scatterplot of cerebroplacental ratio (CPR) measurements across gestational age. Scatterplot illustrating individual CPR values across gestational age from 24 to 36 weeks in the total study population (n = 262). CPR values show wide inter-twin-individual variability without a clear gestational age-related trend within the examined time window.

EFW, UA-PI, MCA-PI, and CPR were similar across all groups (*p* > 0.05). Median CPR values ranged from 1.66 to 1.79, with the highest value observed in the GDM group; however, the difference was not statistically significant (*p* > 0.05). Overall, no statistically significant differences in CPR were observed across the three time points (*p* > 0.05), indicating stable cerebral and placental blood flow dynamics among all groups (Figure 1, Table 2), although these findings should be interpreted cautiously given the exploratory nature of the analysis. Three regression models were used to assess whether CPR depends on its preceding value; no significant association was found.

**Figure 2:**
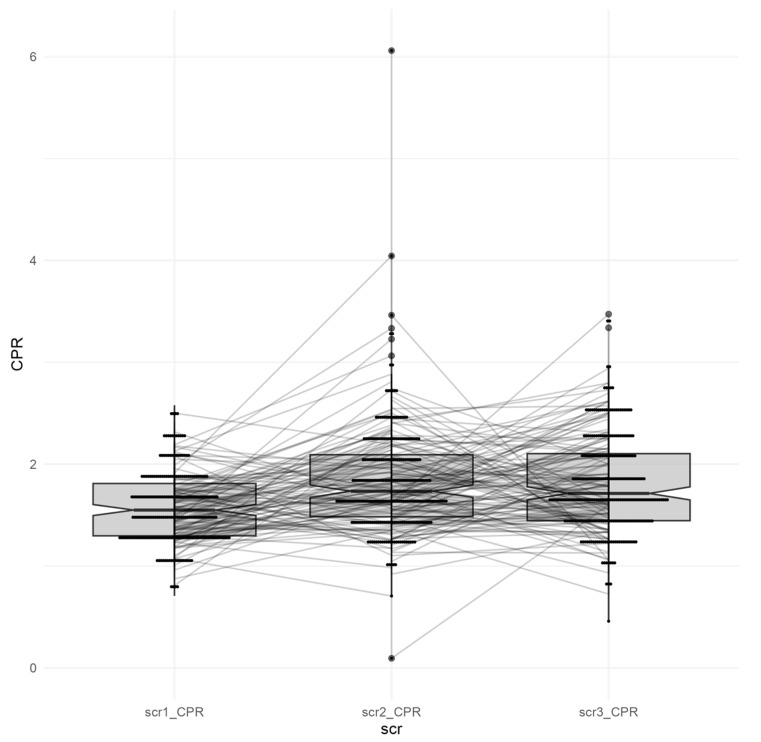
Longitudinal cerebroplacental ratio (CPR) measurements across three antenatal time points in the total study population (n = 262). Boxplots with overlaid individual patient trajectories illustrate CPR values at 24–26 weeks (scr1_CPR), 28–32 weeks (scr2_CPR), and 34–36 weeks (scr3_CPR). Median CPR values remained stable over time with modest variability; no systematic trend or significant change in CPR was observed across gestation in the overall cohort.

Furthermore, analyzing UA-PI and MCA- PI separately in MCDA twin pregnancies with and without (I)GDM did not show significant changes across the three groups as well, suggesting stable placental vascular resistance. While EFW was significantly higher at 28–32 weeks in the GDM and IGDM group, this difference was not observed at later gestation, suggesting a transient growth effect (Figure 2).

**Figure 3:**
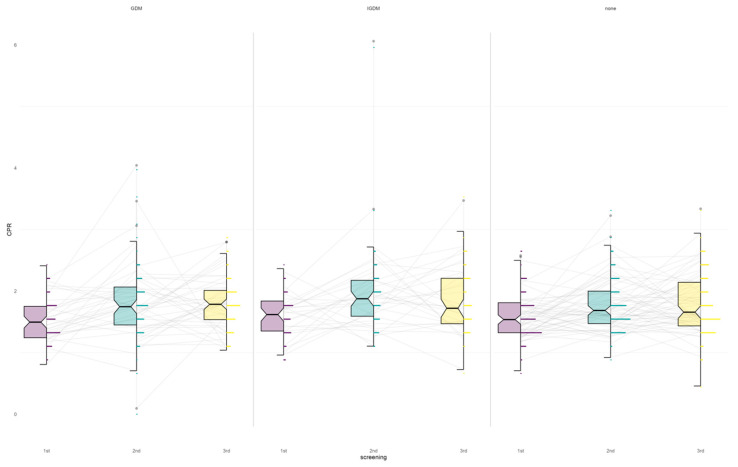
Boxplot of cerebroplacental ratio (CPR) measurements in 1^st^ trimester (purple), 2^nd^ trimester (green) and 3^rd^ trimester (yellow). Longitudinal cerebroplacental ratio (CPR) across three antenatal screenings in pregnancies with gestational diabetes mellitus (GDM, n = 80), insulin-treated GDM (IGDM, n = 62), and non-GDM controls (n = 120). CPR values obtained during three standardized fetal screening time points (1st: 24+0 to 26+6 weeks; 2nd: 28+0 to 32+0 weeks; 3rd: 34+0 to 36+6 weeks) are displayed as boxplots stratified by group (GDM, n = 80; IGDM, n = 62; non-GDM, n = 120). Median, interquartile range, and individual patient trajectories are shown for each group across gestation.

The findings indicate that gestational age at delivery, Apgar and Doppler parameters, and most growth markers remain comparable in MCDA twins with and without (I)GDM. All three groups showed a similar distribution of CPR values at each screening stage, suggesting no major trends between the twin groups (Figure 3).

## 4. Discussion

This study investigated the longitudinal changes in the cerebroplacental ratio in MCDA twin pregnancies, comparing those complicated by GDM and IGDM to those without. The findings indicate that, while (I)GDM is associated with certain maternal and fetal characteristics, no statistically significant differences in CPR measurements were detected throughout gestation. Overall, CPR values remained within normal ranges throughout all screening points (24–26, 28–32, and 34–36 weeks’ gestation) across all three groups. While EFW at 28+0–32+6 weeks of gestation was significantly higher in the IGDM group, we did not observe an effect on CPR or Doppler parameters. Despite these differences in EFW, the lack of significant variation in CPR suggests that the increased fetal size in GDM pregnancies does not necessarily correspond to altered cerebroplacental hemodynamics. This could imply that the compensatory mechanisms in MCDA twin pregnancies effectively maintain fetal cerebral perfusion, even in the presence of GDM-related growth acceleration. However, this interpretation remains speculative, as the study was not designed to directly assess underlying physiological mechanisms. Also, given the exploratory design and absence of adjustment for potential confounders, subtle effects cannot be excluded. Our findings align with a previous study reporting preserved fetal hemodynamics, including CPR, in well-managed singleton pregnancies, complicated by GDM [10].

A recently published systematic review described that CPR can be associated with some adverse perinatal outcomes in women with GDM such as low birthweight, perinatal mortality, and transfer to neonatal unit. However, the diagnostic performance was poor, and therefore the authors do not recommend the use of cerebroplacental ratio as a universal screening for pregnancy complication in women with diabetes [6]. Nevertheless, there are contradictory statements in the literature. This inconsistency highlights the complexity of Doppler assessment in pregnancies affected by metabolic conditions and underscores the need for further research, particularly in twin populations. There is evidence which shows a correlation between low CPR values and growth restriction (<10th percentile) as well as an increased risk of perinatal death and admission to the neonatal unit regardless of type of diabetes [6,11]. In contrast, the study of Familiari et al. supports our results that there is no correlation for CPR and adverse pregnancy outcome [10]. A low CPR may also reflect a failure of a fetus to reach its genetic growth potential at term despite not being classified as small for gestational age [12,13].

Maternal BMI was significantly higher in women with GDM and particularly in those requiring insulin treatment, consistent with known risk factors for GDM [14]. Gestational age at delivery, Apgar scores, and umbilical arterial pH did not differ significantly between groups, indicating that perinatal outcomes were not negatively affected by GDM in this cohort. There are conflicting results in the literature regarding the individual Doppler values and their significance in relation to GDM. As one work states that MCA-PI alone seems to be the best predictor regarding low Agar score, pH and composite adverse outcome [10], another cohort study on the role of CPR in pregnancies with preexisting diabetes and GDM revealed that the ratio is influenced more by the UA-PI rather than the MCA-PI, suggesting that pathogenesis of diabetes may influence the Doppler measurements in the UA-PI more than the MCA [5]. Furthermore, the prognostic accuracy of the UA-PI in pregnancies complicated by diabetes was reported to be superior compared to MCA-PI or CPR [15]. A more detailed analysis of MCA-PI reveals heterogeneous data about MCA-PI and association with pregnancies both with and without GDM. While a recent systematic review showed that there is no significant difference in MCA-PI, some authors report an increased MCA-PI in GDM patients [16,17,18]. This heterogeneity of data illustrates the complexity of Doppler interpretation in pregnancies affected by metabolic disorders.

The absence of significant differences in CPR values across the groups in MCDA twin pregnancies, as demonstrated in this study, do not support a clear association of routine CPR monitoring predicting GDM-related adverse outcomes in twins, unlike its established role in twin-to-twin transfusion syndrome. However, the clinical relevance in this specific high-risk population can still not be excluded. In MCDA twin pregnancies, the single placenta provides uneven blood distribution between the twins, resulting in altered hemodynamic patterns compared to singleton pregnancies. These anastomoses may balance inter-twin hemodynamic changes, thereby stabilizing Doppler indices such as the UA-PI and MCA-PI, and consequently the CPR, even in the context of maternal metabolic disorders such as gestational diabetes [19,20]. These inter-placentar vascular connections may therefore mask the subtle effects of maternal glycemic imbalance, making CPR less responsive or predictive in this population. In singleton pregnancies, GDM—particularly when poorly controlled—is known to influence fetoplacental vascular resistance, sometimes resulting in lower MCA-PI values and a reduced CPR, as reported earlier [10,21]. However, in twins, and especially MCDA twins, the situation is different. Even in the presence of increased fetal growth—as seen in our IGDM group—the expected changes in CPR were not observed, supporting the theory that compensatory mechanisms or the unique vascular physiology in twins modulate the hemodynamic response. Nevertheless, the absence of statistically significant differences in CPR between groups should be interpreted with caution. The study was exploratory in nature, without correction for multiple testing and without multivariable adjustment for baseline differences such as BMI, parity, or assisted conception. Additionally, stratification into three groups and repeated measurements across gestation may have reduced statistical power. Therefore, the absence of statistically significant findings should not be interpreted as evidence of equivalence or absence of an effect.

These findings indicate no detectable group differences in CPR, but do not allow conclusions regarding predictive clinical utility. However, the observed differences in EFW highlight the importance of monitoring fetal growth in GDM pregnancies to manage potential complications associated with macrosomia and should be considered when managing twin pregnancies with diabetes, which has already been described in detail in previous works [3,14,22].

### Strengths and Limitations

Strengths of this study include the relatively large MCDA twin cohort, serial assessment of CPR across pregnancy, and detailed stratification by GDM status. While this study provides valuable insight, certain limitations must be acknowledged. The retrospective design may introduce selection bias, and the sample size, particularly in the IGDM group, may limit the generalizability of the findings as well as the absence of glycemic control parameters (e.g., HbA1c). The absence of glycemic control parameters is a major limitation, as insulin therapy represents only an indirect proxy of disease severity. Another limitation is the lack of adjustment for group differences that have been observed (such as BMI and parity). Overall, the study may be underpowered to detect small but clinically relevant differences, particularly after stratification into three groups and across multiple time points.

## 5. Conclusions

In conclusion, this study demonstrates that while GDM in MCDA twin pregnancies is associated with increased maternal BMI and higher EFW, no statistically significant differences in CPR measurements were observed throughout gestation. CPR values do not significantly differ between MCDA twin pregnancies with and without GDM over gestation. CPR appears to be relatively stable across all time points in all groups. These results indicate that no statistically significant differences in CPR were detected between groups in this retrospective cohort. However, these findings should not be interpreted as evidence of absence of an effect or clinical irrelevance, particularly given the limited statistical power after stratification and the lack of multivariable adjustment. Further studies are needed to assess the prognostic value.

## Figures and Tables

**Table 1:** Baseline characteristics of the study population.

Variable	All (n = 262)	GDM (n = 80)	IGDM (n = 62)	Non-GDM (n = 120)	*p*-Value
BMI (median [IQR])	23.17 [21.08–27.16]	24.57 [21.99–27.64]	25.06 [22.49–28.7]	21.48 [20.2–24.44]	<0.0001
Gravida (median [IQR])	2 [1–3]	1.5 [1–3]	2 [1–2.75]	2 [1–3]	0.7474
Parity (median [IQR])	1 [1–2]	2 [1–3]	2 [1–3]	0 [0–1]	<0.0001
GA at Delivery (weeks)	36.57 [36–36.86]	36.43 [36–36.89]	36.43 [36.18–36.86]	36.57 [36–36.86]	0.8268
APGAR 5 min	10 [9–10]	10 [9–10]	10 [10–10]	10 [9–10]	0.1277
Umbilical Artery pH	7.31 [7.28–7.34]	7.31 [7.28–7.35]	7.3 [7.27–7.33]	7.31 [7.28–7.34]	0.1385
Conception Assisted Spontaneous	44 (16.9%) 216 (83.08%)	14 (17.5%) 66 (82.5%)	18 (29.0%) 44 (70.97%)	12 (10.2%) 106 (89.83%)	0.0065
Smoking	26 (10.0%)	6 (7.5%)	10 (16.1%)	10 (8.5%)	0.1934
Fetal sex Male female	140 (53.4%) 122 (46.56%)	46 (57.5%) 34 (42.5%)	40 (64.5%) 22 (35.48%)	54 (45.0%) 66 (55%)	0.0315

Data are presented as median (interquartile range) or number (percentage). GDM = gestational diabetes mellitus; IGDM = insulin-treated GDM; GA = gestational age; BMI = body mass index. *p*-values were calculated using Kruskal–Wallis test for continuous variables and chi-squared test for categorical variables.

**Table 2:** Doppler measurements and EFW measurements across three time points in pregnancy.

	**1st Screening**			
	EFW (g)	UA-PI	MCA-PI	CPR
All (n = 262)	750 (677–827.75)	1.1 (1–1.22)	1.7 (1.51–1.9)	1.55 (1.3–1.8)
GDM (n = 80)	736.5 (667–822.75)	1.1 (1–1.23)	1.7 (1.44–1.9)	1.5 (1.24–1.8)
IGDM (n = 62)	757.5 (667.7–813.5)	1.12 (1–1.22)	1.79 (1.5–1.9)	1.62 (1.35–1.8)
Non-GDM (n = 120)	752.5 (684.7–839.7)	1.1 (0.99–1.22)	1.7 (1.56–1.9)	1.54 (1.32–1.8)
*p*-value	*p* > 0.05	*p* > 0.05	*p* > 0.05	*p* > 0.05
	**2nd Screening**			
	EFW (g)	UA-PI	MCA-PI	CPR
All (n = 262)	1531 (1373.5–1729.3)	1.1 (1–1.22)	1.8 (1.6–2.0)	1.73 (1.5–2.1)
GDM (n = 80)	1531.5 (1386.8–1672.8)	1.1 (1–1.23)	1.77 (1.57–2)	1.75 (1.5–2.1)
IGDM (n = 62)	1613.5 (1484–1789)	1.12 (1–1.22)	1.81 (1.6–2.1)	1.88 (1.6–2.2)
Non-GDM (n = 120)	1481 (1282.3–1713.8)	1.1 (0.99–1.2)	1.8 (1.6–2.1)	1.69 (1.47–2)
*p*-value	*p* < 0.01	*p* > 0.05	*p* > 0.05	*p* > 0.05
	**3rd Screening**			
	EFW (g)	UA-PI	MCA-PI	CPR
All (n = 262)	2255 (2044–2473)	0.94 (0.82–1.07)	1.59 (1.41–1.8)	1.71 (1.44–2.1)
GDM (n = 80)	2227 (2047–2524)	0.94 (0.82–1.03)	1.62 (1.4–1.76)	1.79 (1.54–2.01)
IGDM (n = 62)	2234.5 (1999–2488)	0.94 (0.78–1.07)	1.6 (1.4–1.9)	1.72 (1.47–2.21)
Non-GDM (n = 120)	2270 (2046–2467)	0.93 (0.83–1.06)	1.58 (1.4–1.77)	1.66 (1.43–2.14)
*p*-value	*p* > 0.05	*p* > 0.05	*p* > 0.05	*p* > 0.05

Diabetes mellitus (GDM, n = 80), insulin-treated GDM (IGDM, n = 62), and non-GDM controls (n = 120). Estimated fetal weight (EFW), umbilical artery pulsatility index (UA-PI), middle cerebral artery pulsatility index (MCA-PI), cerebro-placental ratio (CPR). EFW and Doppler values obtained during three standardized fetal screening time points (1st: 24+0 to 26+6 weeks; 2nd: 28+0 to 32+0 weeks; 3rd: 34+0 to 36+6 weeks). Median and interquartile range for each group across gestation are shown in Table 2.

## Data Availability

The data presented in this study are available upon request from the corresponding author. The data are not publicly available due to data privacy.

## Supplementary Materials

The following supporting information can be downloaded at: https://www.mdpi.com/article/10.3390/jcm15103864/s1, Table S1: CPR Data not available; Table S2: Regression analyses assessing the association between prior CPR measurements and CPR at screening 3.

## Abbreviations

The following abbreviations are used in this manuscript:

BMI	Body mass index
CPR	Cerebroplacental ratio
EFW	Estimated fetal weight
FGR	Fetal growth restriction
GDM	Gestational diabetes mellitus
MCDA	Monochorial diamnial
MCA-PI	Middle cerebral artery pulsatility index
UA-PI	Umbilical artery pulsatility index
TTTS	Twin-to-twin transfusion syndrome
