# Supplementary material for: Cerebroplacental Ratio in Monochorionic Diamniotic Twin Pregnancies with and Without Gestational Diabetes: A Longitudinal Cohort Study
Source: J Clin Med. 2026 May 17;15(10):3864. doi: 10.3390/jcm15103864 (PMC13207628; doi:10.3390/jcm15103864)
Supplement: Supplementary file 1 [file jcm-15-03864-s001.zip › jcm-4228096-supplementary.pdf]

Table S1

	CPR data not available *n* (%)		
	1st Screening	2nd Screening	3rd Screening
**All (*n*=262)**	37 (14.1%)	11 (4.2%)	0
**GDM (*n*=80)**	11 (13.8%)	3 (3.8%)	0
**IGDM (*n*=62)**	10 (16.1%)	4 (6.5%)	0
**Non-GDM (*n*=120)**	16 (13.3%)	7 (3.3%)	0

Table S2. Regression analyses assessing the association between prior CPR measurements and CPR at screening 3.

Model	Variable	*p*-value
1.1 CPR at screening 3 ~ CPR at screening 1	CPR	0.5023
	CPR (nonlinear)	0.5631
	CPR × GA at screening	0.1725
	DM (yes/no)	0.2323
	**Entire model**	0.2415
1.2 CPR at screening 3 ~ CPR at screening 2	CPR	0.4446
	CPR (nonlinear)	0.2969
	CPR × GA at screening	0.6228
	DM (yes/no)	0.5798
	**Entire model**	0.0015
1.3 CPR at screening 3 ~ CPR at screening 1 + 2	CPR screening 1	0.6628
	CPR screening 1 (nonlinear)	0.7977
	CPR screening 2	0.5939
	CPR screening 2 (nonlinear)	0.4536
	CPR × GA at screening 1	0.5760
	CPR × GA at screening 2	0.1513
	(CPR screening 2 / screening 1) × GA	0.1172
	DM (yes/no)	0.3487
	**Entire model**	0.0048

Across all three regression models, prior CPR measurements (screenings 1 and/or 2) were not significantly associated with CPR at screening 3.
